# Delayed Imitation of Lipsmacking Gestures by Infant Rhesus Macaques (*Macaca mulatta*)

**DOI:** 10.1371/journal.pone.0028848

**Published:** 2011-12-12

**Authors:** Annika Paukner, Pier F. Ferrari, Stephen J. Suomi

**Affiliations:** 1 Laboratory of Comparative Ethology, NIH Animal Center, Poolesville, Maryland, United States of America; 2 Dipartimento di Neuroscienze and Dipartimento di Biologia Evolutiva e Funzionale, Universita di Parma, Parma, Italy; French National Centre for Scientific Research, France

## Abstract

Human infants are capable of accurately matching facial gestures of an experimenter within a few hours after birth, a phenomenon called neonatal imitation. Recent studies have suggested that rather than being a simple reflexive-like behavior, infants exert active control over imitative responses and ‘provoke’ previously imitated gestures even after a delay of up to 24 h. Delayed imitation is regarded as the hallmark of a sophisticated capacity to control and flexibly engage in affective communication and has been described as an indicator of innate protoconversational readiness. However, we are not the only primates to exhibit neonatal imitation, and delayed imitation abilities may not be uniquely human. Here we report that 1-week-old infant rhesus macaques (*Macaca mulatta*) who show immediate imitation of a lipsmacking gesture also show delayed imitation of lipsmacking, facilitated by a tendency to refrain from lipsmacking toward a still face during baseline measurements. Individual differences in delayed imitation suggest that differentially matured cortical mechanisms may be involved, allowing some newborns macaques to actively participate in communicative exchanges from birth. Macaque infants are endowed with basic social competencies of intersubjective communication that indicate cognitive and emotional commonality between humans and macaques, which may have evolved to nurture an affective mother-infant relationship in primates.

## Introduction

Neonatal imitation, the phenomenon that newborn human infants can accurately match facial gestures, was reported over 30 years ago [1], and remains a thriving research topic within the social sciences. Part of the continued interest in this phenomenon lies in the fact that from the start, it was clouded in controversy. While several investigators confirmed the first findings [2]–[4], others failed to replicate the same effects [5]–[7]. The main criticisms appear to be leveled at the range of gestures that are reliably matched, as well as the interpretation of this phenomenon as imitation. For example, in a review of studies Anisfeld [8], [9] purported that infants only match tongue protrusions, and that imitation of only one gesture is more parsimonious with an arousal explanation rather than an imitation interpretation [10]–[12]. Herein lies the crux of many disagreements – while the phenomenon that neonates can match adult behaviors under certain circumstances is generally accepted [11], the big question still being asked is whether this type of behavioral matching should be described as imitation. Generally speaking, imitation can be concluded when the action of a model is matched, where matching entails a causal relation as well as similarity of modeled and imitated action [13], [14]. Laboratory assessments generally infer imitation when the frequency of a target behavior after modeling is greater than the frequency of the same behavior in a control condition. This conceptualization is purely based on behavior, and does not address issues such as intentionality, uniqueness, novelty, or generalizability of the imitated gesture. As Heimann, page 74, [15] writes, “Viewed in this way, there is no doubt that neonatal imitation is a real phenomenon. It does exist and it can be demonstrated as has been shown by numerous research groups”.

Coupled to the issue of whether the phenomenon should be called imitation is uncertainty about the function of neonatal imitation. While some consider neonatal imitation to be a simple reflexive-like phenomenon [16], others have proposed that infants can exert active control over imitative responses and ‘provoke’ previously imitated gestures [17]. This latter hypothesis implies that during imitative episodes, infants do not just automatically respond to the caregiver's stimulation, but that they can also flexibly sustain imitative exchanges and show voluntary control over their actions. The capacity to imitate and to engage in flexible turn-taking behaviors is a developmental landmark for early forms of communication and has been proposed to play a crucial role in facilitating an affective connection with the caregiver [18], [19].

Delayed imitation, copying a gesture or an action after a delay, can be regarded as a signature of the transition toward a more sophisticated capacity to control and flexibly engage in communication. There is evidence that human infants are capable of delayed imitation of object-directed actions starting at 6 months of age [20] and of facial gestures at 6 weeks of age [21]. Given the complexity of the cognitive skills that are required to express delayed imitation, it is presently unclear whether it is a uniquely human feature or whether it could also be present in other primates. Recent evidence shows that we share certain neonatal imitation abilities with some of our primate relatives including chimpanzees [13], [22] and rhesus macaques [23]–[25], but to date there has been no investigation of delayed neonatal imitation in non-human primates.

In the present study, we tested whether rhesus macaque infants could show delayed imitation of a facial gesture. We focused on one gesture only, lipsmacking, because this facial gesture is likely to carry the most communicative meaning for macaques [26] and may therefore be most easily imitated. Moreover, rather than looking at delays of hours or a day [21], we tested infants after a delay of only 1 minute, which may more closely resemble a naturalistic communicative situation between macaque mothers and their infants. We suggest that increases in the frequency of LPS gestures after seeing a human experimenter demonstrate LPS gestures compared to a still face baseline, the increase being larger in the lipsmacking condition than in other control conditions, can be seen as evidence of neonatal imitation. Our results indicate that rhesus macaque infants who show immediate imitation of lipsmacking gestures also show imitation after a 1 minute delay.

## Results

**Figure 1:**
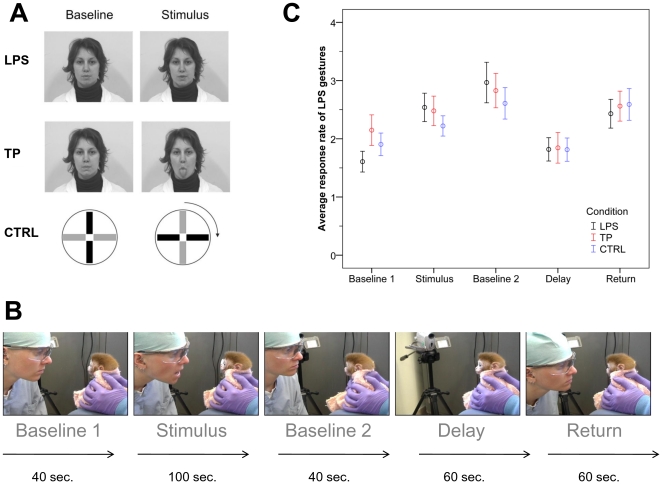
Illustration of the experimental conditions and LPS responses for all infants. A. Illustration of modeled gestures. LPS: lipsmacking. TP: tongue protrusion. CTRL: control condition in which a disk was presented in front of the infant during the baseline period. During the stimulus period, the disk was rotated both clock and counter-clockwise. B. Illustration of an example LPS trial with durations of each phase. C. Average response rates of LPS per 40 sec +/− SEM in LPS, TP, and CTRL conditions for all infants (N = 60) across time periods.

Sixty infant rhesus macaques (*Macaca mulatta*) were reared from birth in a nursery facility and were tested three times a day for up to four days in their first week of life (see Materials and Methods). We presented three different stimuli to infants: a lipsmacking gesture (LPS, rapid opening and closing of the mouth), a tongue protrusion gesture as a facial motion control condition (TP, protrusion and retraction of the tongue), and a non-biological control condition (CTRL; a white plastic disk with orthogonal black and red stripes was slowly rotated left and right; see also Figure 1A). At the beginning of a trial, a 40-sec baseline was conducted, in which the demonstrator displayed a passive/neutral facial expression (or still disk in CTRL). The demonstrator then displayed a facial gesture (LPS or TP, or rotating disk in CTRL) for 20 seconds, followed by a still face (still disk in CTRL) period for another 20 sec. This stimulus-still face sequence was repeated three times, with the last still face period lasting 40 sec. The demonstrator then stood up and walked behind the experimenter holding the infant, thereby removing himself/herself from the infant's visual field. Infants continued to be held by the first experimenter and were kept facing forward but without any particular visual focus, for 60 sec. After this delay period, the demonstrator returned to his/her initial position in front of the infant, and displayed a still face/neutral expression (still disk in CTRL) for another 60 sec (see also Figure 1B).

For analysis, we divided each trial into 5 time periods: the first 40 sec still face/disk phase prior to any stimulus presentation (Baseline 1); the period starting with the first stimulus presentation and ending with the third stimulus presentation (Stimulus); the second 40 sec still face/disk phase following the third stimulus presentation (Baseline 2); the 60 sec delay period in which no stimulus was displayed (Delay); and finally, the 60 sec still face/disk phase following the delay period (Return). We analyzed the progression of performed LPS behaviors during these 5 time periods within each condition and across conditions. We hypothesized that if infants imitate LPS gestures, we would see an increase between Baseline 1 and Stimulus, which would be larger in the LPS condition compared to the control conditions. Moreover, if infants initiate LPS responses after a 1 minute delay, we would see an increase in LPS responses between Baseline 1 and Return, which would also be larger in the LPS condition compared to the control conditions.

### Imitation and delayed imitation in all infants

In order to investigate whether LPS gestures would increase in response to seeing LPS gestures being performed by the model, we first analyzed data within the LPS condition using a repeated measures ANOVA, which showed a significant effect for time period (F(4, 236) = 9.40, p<0.001). Post-hoc comparisons revealed a significant increase in LPS responses between Baseline 1 to Stimulus (1.6 to 2.5, p<0.001), no difference between Stimulus and Baseline 2 (2.5 to 3.0, p = 0.48), a significant decrease between Baseline 2 and Delay (3.0 to 1.8, p<0.001), and a significant increase between Delay and Return (1.8 to 2.4, p = 0.008). Moreover, levels of LPS responses during Baseline 1 were significantly different from levels in all other time periods (all p<0.001) with the exception of the Delay period, in which levels of LPS responses did not differ (p = 0.18). In order to confirm previous findings [23], we then investigated whether the increase in LPS responses between Baseline 1 and Stimulus was specific to the LPS condition. A repeated measures ANOVA with time period (2) and condition (3) as within-subject factors was run, which revealed a significant main effect for time period (F(1, 59) = 27.33, p<0.001) modified by an interaction (F(2, 118) = 3.72, p = 0.027) but no effect for condition (F(2, 118) = 0.67, p = 0.52). Contrast analyses showed that lipsmacking responses increased more sharply in the LPS condition (1.6 to 2.5) than in the TP condition (2.1 to 2.5, p = 0.03) or the CTRL condition (1.9 to 2.2, p = 0.028). The same analysis using Baseline 1 and Return as time periods showed an effect for time (F(1, 59) = 15.32, p<0.001), but no effect for condition and no interaction (both p>0.05). Increases in levels of LPS responses from Baseline 1 to Return did not differ significantly between conditions (see also Figure 1C).

### Individual differences: imitators and non-imitators

Even though infants showed an imitation effect for LPS gestures as a group, not all infants responded strongly to the presentation of LPS gestures. Some infants appeared to respond during the Stimulus phase just as much as during Baseline 1, and others even reduced the frequency of responses during the Stimulus phase. Similar variability in neonatal imitation has previously been observed in human infants [2], [27] and macaque infants [25]. To capture some of these individual differences, we compared the difference in LPS gestures between Stimulus and Baseline 1 in the LPS condition and in the CTRL condition. We classified infants as imitators if (i) Infants increased their rate of responding during the Stimulus phase, i.e. the difference between Stimulus and Baseline 1 in the LPS condition was larger than zero (0), and (ii) The difference between Stimulus and Baseline 1 was larger in the LPS condition than in the CTRL condition. Using these criteria, 33 infants were classified as imitators and 27 infants were classified as non-imitators, a similar proportion of imitators as found in human infants for mouth opening imitations (2). We then proceeded to analyze the data for the two groups separately (imitator and non-imitator).

### Imitation and delayed imitation in imitators

**Figure 2:**
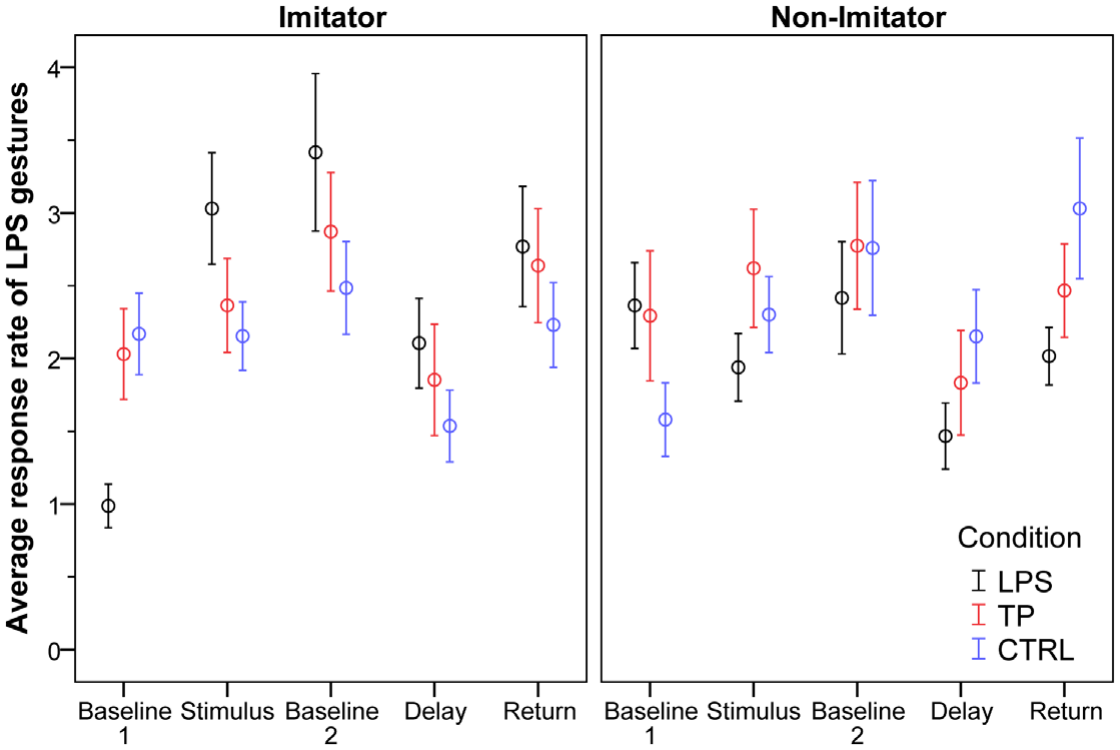
Average response rates of LPS per 40 sec +/− SEM broken down into Imitators (left, N = 33) and Non-imitators (right, N = 27) in LPS, TP, and CTRL conditions across time periods.

For imitators, analysis of LPS responses within the LPS condition showed a significant effect for time period (F(4, 128) = 16.90, p<0.001). Post-hoc comparisons showed that Baseline 1 differed significantly from Stimulus (1.0 to 3.0, p<0.001), Stimulus did not differ from Baseline 2 (3.0 to 3.4, p = 0.79), Baseline 2 differed significantly from Delay (3.4 to 2.1, p = 0.005), and Delay did not differ significantly from Return (2.1 to 2.8, p = 0.011). In addition, Baseline 1 differed significantly from Return (1.0 to 2.8, p<0.001). Comparing across conditions, there was no main effect for condition (F(2,64) = 0.46, p = 0.64) but a main effect for time (F(4, 128) = 13.08, p<0.001) and an interaction between condition and time (F(8, 256) = 6.89, p<0.001). Contrast analyses further revealed that the difference between Baseline 1 and Stimulus was significantly greater in the LPS condition (1.0 to 3.0) than in the TP condition (2.0 to 2.4, p<0.001) or the CTRL condition (2.2 to 2.2, p<0.001). Finally, the difference between Baseline 1 and Return was also significantly greater in the LPS condition (1.0 to 2.8) than in the TP condition (2.0 to 2.6; p<0.001) and the CTRL condition (2.2 to 2.2, p<0.001; see also Figure 2).

### Imitation and delayed imitation in non-imitators

For non-imitators, there was a marginal main effect for time period for LPS responses within the LPS condition (F(4, 104) = 2.44, p = 0.051). Post-hoc comparisons showed that there was a significant drop in responses between Baseline 2 and Delay (2.4 to 1.5, p = 0.023), and an increase between Delay and Return (1.5 to 2.0, p = 0.031). No other differences between consecutive time periods were found. In addition, the difference between Baseline 1 and Return was not significant (2.4 to 2.0, p = 0.63). Looking at responses across conditions, there was a main effect for time (F(4, 104) = 3.68, p = 0.012) but no effect for condition and no interaction (both p>0.05). Post-hoc comparison showed a general drop in responses between Baseline 2 and Delay (p = 0.004) and a general increase in responses between Delay and Return (p = 0.04; see also Figure 2).

### Delayed imitation: comparing imitators with non-imitators

Since imitators and non-imitators were classified according to their performance during Baseline 1 and Stimulus, it is no surprise that the two groups differed in their performance during these time periods. It also appears that imitators, but not non-imitators, respond more strongly during the Return phase compared to Baseline 1. To directly test whether there was a difference between the two groups, we ran a further repeated measures ANOVA on the mean difference between Baseline 1 and Return with condition (3) as within-subject factor and imitator (2) as between subject factor. This analysis revealed no effect for condition (F(2, 116) = 3.18, p = 0.07) or for imitator (F(1, 58) = 0.55, p = 0.46), but an interaction between condition and imitator (F(2, 116) = 8.19, p = 0.004). Post-hoc comparisons showed that imitators showed a significantly greater increase in LPS responses during the LPS condition than non-imitators (mean imitators: 1.8, mean non-imitators: −0.3, p<0.001), but that non-imitators showed a significantly greater increase in LPS responses in the CTRL condition than imitators (mean imitators: 0.1, mean non-imitators: 1.5, p = 0.044). No differences between imitators and non-imitators were found for the TP condition (p = 0.95).

### Baseline measures: imitators and non-imitators

The imitation effect appears to be facilitated by two congruent factors: in the LPS condition, imitators responded with particular high frequencies of lipsmacking during the Stimulus period, but also showed particularly low responses of lipsmacking during Baseline 1. Even though as a group, there was no significant differences in LPS responses in Baseline 1 of all three conditions (F(2, 118) = 2.13, p = 0.12) or the Stimulus period (F(2, 118) = 0.79, p = 0.46), when factoring imitator status into the ANOVAs, we found a significant imitator*condition interaction for Baseline 1 (F(2, 116) = 11.52, p<0.001) as well as for Stimulus (F(2, 116) = 4.85, p = 0.009). Post-hoc comparisons indicated that imitators showed significantly fewer LPS responses than non-imitators during Baseline 1 of the LPS condition (1.0 vs. 2.4, p<0.001), and significantly higher LPS responses than non-imitators during Stimulus of the LPS condition (3.0 vs. 1.9, p = 0.018). The same analysis for LPS responses in the Return phase failed to find any significant effects (all p>0.05), indicating that during the Return phase, imitators as well as non-imitators responded at similar LPS frequencies in all conditions. To investigate whether the differences during Baseline 1 and Stimulus might have developed over the four test sessions, we compared LPS responses during Baseline 1 and Stimulus from imitators and non-imitators who had completed all four test sessions (imitator N = 26, non-imitator N = 17). For Baseline 1, an ANOVA with test session (4) as within-subject factor and imitator (2) as between subject factor showed no effect for test session (F(3, 123) = 0.55, p = 0.65) but an effect for imitator (F(1, 41) = 8.40, p = 0.006), and no interaction (F(3, 123) = 0.34, p = 0.80). Imitators showed consistently lower LPS frequencies during Baseline 1 compared to non-imitators. For Stimulus, an ANOVA showed no effect for test session (F(3, 123) = 1.21, p = 0.31) but an effect for imitator (1, 41) = 10.68, p = 0.002) and an interaction (F(3, 123) = 4.41, p = 0.006). Imitators showed higher frequencies of LPS responses on all test days, but particularly on D1 (3.7 vs. 1.4, p = 0.008) and D7 (3.8 vs. 0.7, p<0.001; see also Figure S1).

### LPS vs. TP responses within the LPS condition

Not only did infants respond with LPS gestures, they also performed TP responses in all conditions. We therefore investigated whether infants specifically matched LPS gestures during the LPS condition, or whether they increased both LPS and TP gestures in response to seeing LPS gestures. We conducted a repeated measures ANOVA with time (5) and response (2) as within-subject factors. Imitators did not show a significant effect for response (F(1,32) = 2.76, p = 0.11) but a significant main effect for time (F(4, 128) = 12.13, p<0.001) and a significant interaction between response and time (F(4, 128) = 13.18, p<0.001). Contrast analyses showed that there was a significantly larger increase in LPS responses from Baseline 1 to Stimulus (1.0 to 3.0) than in TP responses (3.1 to 3.1; p<0.001). Moreover, the difference between Baseline 1 and Return was larger for LPS responses (1.0 to 2.8) than for TP responses (3.1 to 2.8, p<0.001). For non-imitators, the same 2(response) by 5 (time) repeated measures ANOVA failed to show any main effects, nor an interaction (all p>0.05; see also Figure S2).

### Baseline measures: Delayed imitation after 24 h?

Previous studies found that at 6 weeks old, human infants will imitate facial gestures after delays of 24 h [21]. Even though the primary aim of the present study was to investigate imitation after a 1 minute delay in infant macaques, our study design allowed us to also evaluate infant macaques' potential imitative abilities after longer delays. Since infants were tested 4 times during the first week of life with individual test sessions falling on one out of two days (determined by other experimental constraints and experimenter availability), we identified those infants who received two test sessions within 24 h of each other. Of those, 13 infants qualified for test sessions on D2–D3, 8 infants qualified for test sessions on D4–D5, 7 infants qualified for test sessions on D6–D7, and 3 infants qualified for test sessions on D2–D3 and D6–D7 (total N = 31, 18 imitators and 13 non-imitators). If infants could imitate LPS responses after 24 h, we hypothesized that in the LPS condition, LPS responses during Baseline 1 would be significantly higher on the second test day compared to LPS responses during Baseline 1 on the previous test day. However, paired samples t-tests indicated that this was not the case (imitators: means 0.89 and 0.89; non-imitators: means 2.31 and 2.54, both p>0.05; Table 1).

**Table 1:** Average increase of LPS responses for a subset of imitators (N = 18) and non-imitators (N = 13).

	Immediate Imitation	1 Minute Delayed Imitation	24 h Delayed Imitation
**Imitator**	1.87 (1.82)	1.85 (2.16)	0 (1.22)
**Non-Imitator**	0.71 (1.05)	−0.65 (1.51)	−0.23 (4.88)

Standard deviations are given in parentheses.

Immediate Imitation = average increase between Baseline 1 and Stimulus from all test days.

1 Minute Delayed Imitation = average increase between Baseline 1 and Return from all test days.

24 h Delayed Imitation = average increase between Baseline 1 and Baseline 1 when two test days were 24 h apart.

## Discussion

Similar to previous reports [23], [25], as a group infant rhesus macaques increased the rate of lipsmacking (LPS) behavior in response to seeing LPS gestures by a human model, and they did so at a higher rate in the LPS condition compared to both control conditions. This phenomenon has been termed ‘neonatal imitation’ in the human [1], [2], [28] as well as the comparative literature [13], [22]–[25]. Within the LPS condition, infants increased LPS during the Stimulus phase, and the frequency of LPS remained high during Baseline 2 when the model displayed a still face. A similar phenomenon in the human infant literature has been termed ‘provocation’ [17], and is thought to be based on infants' desire to sustain an interaction. Furthermore, LPS responses dropped significantly once the model was removed from the infants' visual field during the Delay period, and did not differ from Baseline 1 levels. Such a pattern makes it unlikely that infants' performance was based on an innate releasing mechanism that assumes that once LPS is triggered, it is performed indiscriminately of the environmental circumstances [1], [21]. Infants also increased LPS responses in the Return phase compared to Delay and Baseline 1, however that increase did not differ between conditions.

It was clear from the present data that imitation is not a unitary phenomenon. Taking into account individual differences, we found that infants who were judged to perform immediate imitation of LPS showed consistently lower levels of LPS during Baseline 1 as well as exceptionally high levels of LPS during the Stimulus phase. Imitators also performed higher rates of LPS gestures after a 1-minute delay compared to Baseline 1, and this increase during Return was higher in the LPS condition compared to both control conditions. Moreover, tongue protrusion responses in the LPS condition did not show a similar pattern: in imitators, the observed increase between Baseline 1, Stimulus and Return did not occur for tongue protrusions. However, unlike human infants, there were no indications that imitators were able to match lipsmacking after a delay of 24 h, suggesting that while rhesus macaque infants can tolerate some delay, the window is much smaller than in human infants.

It is worth noting that what determines the labels ‘imitator’ and ‘non-imitator’ here is a description of a relative matching ability, not an absolute response criterion. Absolute levels of LPS responses were similar for imitators and non-imitators in all conditions during the Return phase, perhaps casting doubt on the idea that what we observed can justifiably be called ‘delayed imitation’. It appears that the reported delayed imitation effect was primarily due to exceptionally low responses during Baseline 1 of the LPS condition, effecting the significant increase in response rates. At this time, we do not know why imitators showed these low levels of responding during Baseline 1 of the LPS condition but not the other conditions. Our research design is the first to reveal such a difference in baseline responses, having separate baseline measurements in all conditions. More commonly, developmental researchers first perform one baseline measurement, then present the different conditions in random order [1], [2]. It is possible that the observed low response rate was a random chance occurrence, and had baseline LPS levels in the LPS condition been higher, the corresponding response rate during the Return phase could have been equally increased. It is also possible that the low level of responding is associated with functional significance. Imitators may not spontaneously initiate LPS towards a still face, but once prompted, may be highly responsive to LPS, which would suggest that there is differential sensitivity to social stimuli between imitators and non-imitators. Future studies could evaluate this hypothesis directly. Is the low Baseline 1 response rate a confounding factor for the current results and negates their interpretation of delayed imitation? We believe that since the criteria for delayed imitation (i.e. significant increase between Baseline 1 and Return, specific to the LPS condition) are fulfilled, a conclusion of delayed imitation is acceptable, albeit perhaps cautiously and pending future replication of these results.

We have some indications of what might contribute to immediate imitation abilities. Previous studies with human infants found that there are significant correlations between immediate and delayed imitation performance in typically developing children as well as in children with autism [29]. It appears that both tasks have mechanisms in common, specifically those required to match the behavior of others. The capacity to match one's own with others' behavior appears to rely on a mirror neuron mechanism, which has been found in the macaque ventral premotor and posterior parietal cortices. Mirror neurons are activated during the observation of an action by triggering an internal motor representation of the same action [30]–[32] and thus can directly influence an individual's motor output, as recently demonstrated in adult macaques [32]. Individual differences between infant macaque imitators and non-imitators in patterns of motor development related to cortical motor control of intentional movements have also been reported previously [25]. These results suggest that shortly after birth, imitators seem to have a cortical system better tuned to social stimuli and a more developed capacity to control intentional movements than newborns with poor self-other behavioral matching abilities.

Our findings contribute to clarifying the nature of neonatal imitation in rhesus monkeys. The delayed imitative responses of newborn monkeys argue against a reflex-like phenomenon or, in the old ethological tradition, a stimulus-triggered fixed action pattern. We cannot exclude that viewing facial expressions might trigger matched motor programs. However, the modulation of these programs is complex and not simply stimulus-driven as evidenced by our results. There are recent reports of facial mimicry and contagion in nonhuman primates in which the involuntary nature of the response seems to prevail [33]. However, during delayed imitation, the voluntary nature of the neonatal imitation behavior seems to predominate. During the Return phase, the human face is immobile and the newborn does not receive input from the model about the matched motor program. Mirror activation alone therefore cannot account for this phenomenon, and thus a more complex brain network in addition to mirror neurons is probably required.

From a functional perspective, delayed imitation represents an important developmental landmark because it reveals that in adult-infant relationships, the newborn is not a passive recipient but is actively engaged in intersubjective exchanges with the capacity to promote affective connections through face-to-face engagement [18]. Recently, the presence of early forms of intersubjectivity in primate species other than humans has been documented based on mother-infant synchronous facial exchanges and turn-taking behavior [13], [24]. It is likely that these behaviors represent an important cornerstone for early forms of communication that might not only promote affective bonding between mothers and infants but might also help to regulate infants' emotional and cognitive development.

## Materials and Methods

### Subjects

Subjects were 60 infant rhesus macaques (*Macaca mulatta*), 31 male and 29 female. All infants had been carried to term and had been born without further complications; birth weights fell within normal parameters. All infants were separated from their mothers on the day they were born, and were reared in a nursery facility for ongoing, unrelated research studies. Infants were individually housed in incubators (51 cm×38 cm×43 cm) for the first two weeks of life and in metal cages thereafter. Both housing arrangements contained an inanimate surrogate mother covered with fleece fabric as well as loose pieces of fleece fabric and various rubber toys. For the first month of life, infants could see and hear, but not physically contact, other infants of similar age. For further details regarding rearing practices, see [23], [34].

### Procedure

Infants were tested three times a day for up to four days when 1–2 days old, 3–4 days old, 5–6 days old, and 7–8 days old with an interval of at least 1 h between test sessions each day (43 infants were tested on 4 days, 13 infants were tested on 3 days, and 4 infants were tested on 2 days). We presented three different stimuli to infants: a lipsmacking gesture (LPS, rapid opening and closing of the mouth), a tongue protrusion gesture as a facial motion control condition (TP, protrusion and retraction of the tongue), and a non-biological control condition (CTRL; a white plastic disk with orthogonal black and red stripes was slowly rotated left and right). Each stimulus was presented once a day to infants; the order of stimulus presentations remained the same for each infant but was randomized between infants. In each test session, one experimenter held the infant swaddled in pieces of fleece fabric. A second experimenter served as the source of the stimuli, and a third experimenter videotaped the test session (using a Sony Digital Video camcorder ZR600) and ensured correct timing of the different phases of the trial. Individual demonstrators were randomly assigned to conditions but remained consistent within each infant. At the beginning of a trial, a 40 sec baseline was conducted, in which the demonstrator displayed a passive/neutral facial expression (or the still disk in CTRL). The demonstrator then displayed a facial gesture (LPS or TP, or rotating the disk in CTRL) for 20 seconds, followed by a still face (still disk in CTRL) period for 20 sec. This stimulus-still face sequence was repeated three times, however the last still face period was 40 sec long. The demonstrator then stood up and walked behind the experimenter holding the infant, thereby removing himself/herself from the infant's visual field. Infants continued to be held by the first experimenter and were kept facing forward towards the camera, but without any particular visual focus, for 60 sec. After this delay period, the demonstrator returned to his/her initial position in front of the infant, and displayed a still face/neutral expression (still disk in CTRL) for another 60 sec (total trial length: 5 minutes).

### Analysis

Tapes were analyzed using all occurrence sampling of all lipsmacking and tongue protrusion behaviors in each condition and each phase of trials. 19 percent of all tapes were analyzed by a second coder for LPS responses, and 18 percent of LPS tapes were analyzed by a second coder for TP responses; agreement between both coders was high (Pearson correlations: r = 0.81 for LPS and r = 0.92 for TP, both p<0.001). All coders were blind to the experimental conditions. For analysis, we averaged data from all test days and adjusted data of each phase to a common time frame (40 sec) to control for the different lengths of the trial phases. Due to non-normal distributions, all data were square root transformed prior to analysis. Where a Mauchly's test of sphericity indicated a violation of sphericity for repeated measures ANOVAs (p<0.05), Huynh-Feldt adjustments were used.

## Supporting Information

Figure S1 Average response rates of LPS per 40 sec +/− SEM during Baseline 1 (top) and Stimulus (bottom) of Imitators and Non-imitators in the LPS condition across 4 testing days.
(TIF)

Figure S2 Average response rate of LPS and TP per 40 sec +/− SEM during the LPS condition for Imitators (top) and Non-imitators (bottom).
(TIF)
