# Supplementary figures and images for: Delayed Imitation of Lipsmacking Gestures by Infant Rhesus Macaques (Macaca mulatta)
Source: PLoS One. 2011 Dec 12;6(12):e28848. doi: 10.1371/journal.pone.0028848 (PMC3236225; doi:10.1371/journal.pone.0028848)

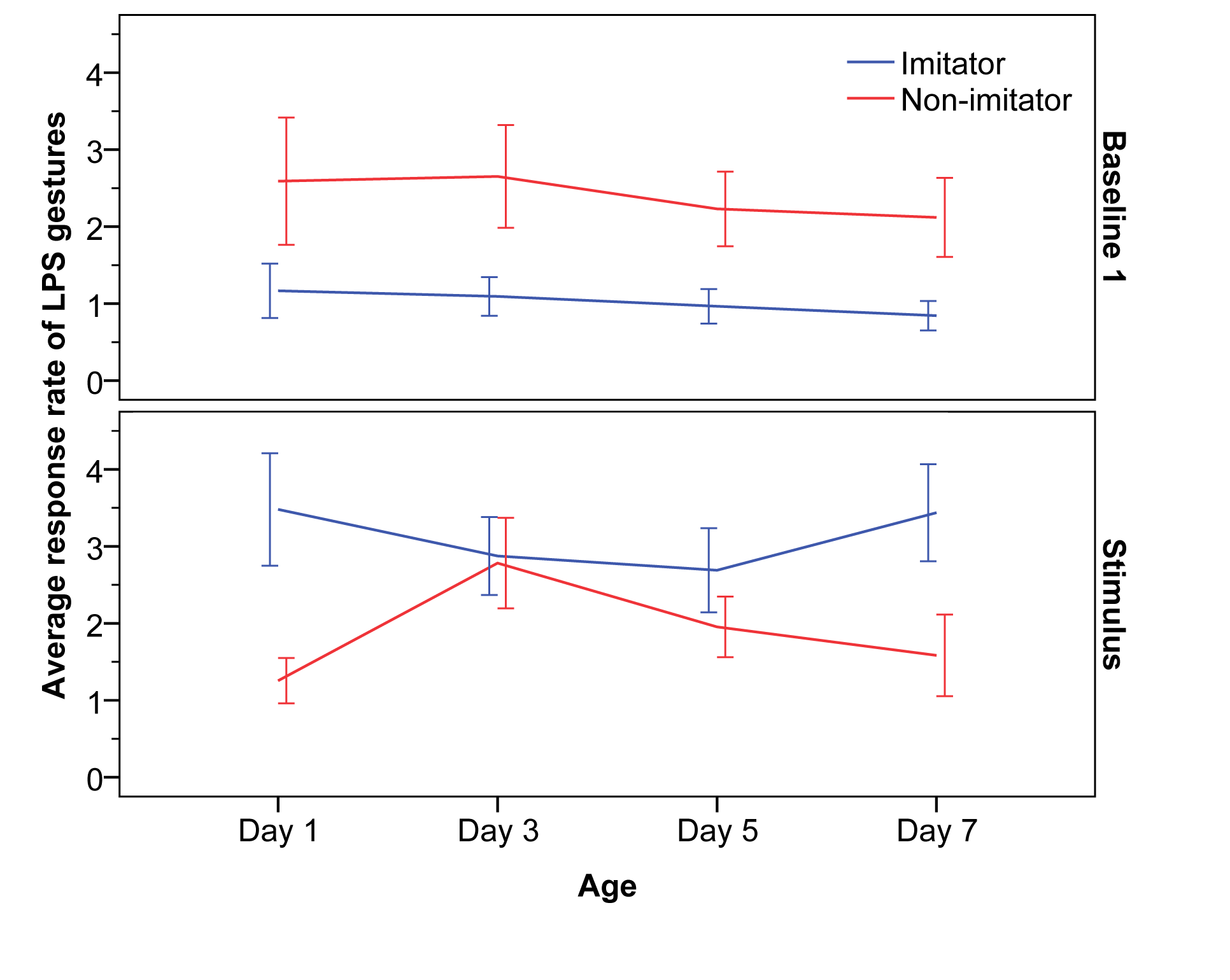

Supplement: Figure S1 — Average response rates of LPS per 40 sec +/− SEM during Baseline 1 (top) and Stimulus (bottom) of Imitators and Non-imitators in the LPS condition across 4 testing days. (TIF) [file pone.0028848.s001.tif]

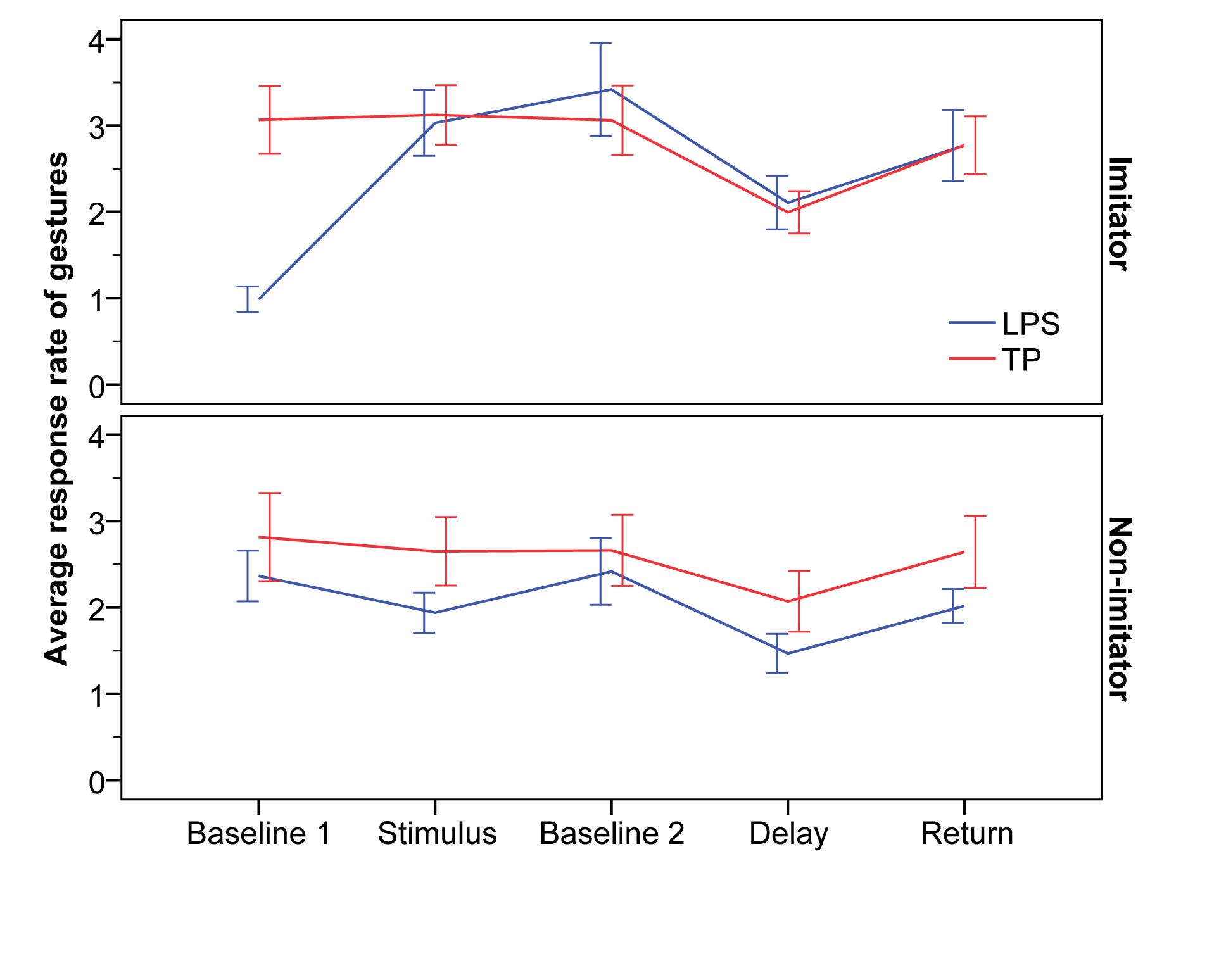

Supplement: Figure S2 — Average response rate of LPS and TP per 40 sec +/− SEM during the LPS condition for Imitators (top) and Non-imitators (bottom). (TIF) [file pone.0028848.s002.tif]
